# The Expression Dynamics of piRNAs Derived From Male Germline piRNA Clusters and Retrotransposons

**DOI:** 10.3389/fcell.2022.868746

**Published:** 2022-05-11

**Authors:** Masaki Kawase, Kenji Ichiyanagi

**Affiliations:** Laboratory of Genome and Epigenome Dynamics, Department of Animal Sciences, Graduate School of Bioagricultural Sciences, Nagoya University, Nagoya, Japan

**Keywords:** piRNA biogenesis, spermatogenesis, PIWI, retrotransponson, FACS, germ cell

## Abstract

In mammals, germ cells produce a class of small regulatory RNAs called PIWI-interacting RNAs or piRNAs, which are 25–32 nucleotides in length. The profile of testicular piRNAs changes during development. The piRNAs detected in fetal testes at embryonic day 13.5 and later are called fetal piRNAs. The piRNAs detected in testes in a period where germ cells do not yet enter the pachytene stage of meiotic prophase I are called pre-pachytene piRNAs, whereas those in testes at later postnatal days are called pachytene piRNAs. Here, to elucidate the exact expression dynamics of these piRNAs during development, we compared piRNAs present in male germ cells at different stages, which were purified by fluorescence-activated cell sorting, and those in embryonic testes. The analysis identified three distinct groups of piRNA clusters: prospermatogonial, early, and late clusters. piRNA length was largely correlated with the repertoire of PIWI-like proteins in respective germ cells; however, the late piRNA clusters tended to generate longer (PIWIL1-type) piRNAs, whereas the early clusters tended to generate shorter (PIWIL2-type) piRNAs, suggesting a cluster- or sequence-dependent mechanism for loading onto PIWI-like proteins. Retrotransposon-derived piRNAs, particularly evolutionary young retrotransposons, were abundantly produced in prospermatogonia, however, their abundance declined as development proceeded. Thus, in later stages, retrotransposon-derived piRNAs were not enriched with those from evolutionary young elements. The results revealed that, depending on the piRNA clusters from which they are derived, longer PIWIL1-type piRNAs are produced earlier, and shorter PIWIL2-type piRNAs remain in a longer period, than previously thought.

## Introduction

PIWI-interacting RNAs (piRNAs) are a class of small regulatory single-stranded RNAs specifically expressed in the gonads of animals, including insects, nematodes, and mammals. In mice, the deficiency in genes involved in piRNA biogenesis, such as *Piwil2* (also called *Mili*), *Piwi4* (*Miwi2*), *Ddx4* (*Mvh*), and *Pld6* (*Miotopld*) leads to retrotransposon de-repression in germ cells and male infertility (Chuma and Nakano, 2013), indicating the important role of piRNAs in male germ cell development and the maintenance of genomic integrity. In male germ cells in mice, piRNAs are predominantly generated from long precursor RNAs transcribed in >200 genomic regions called piRNA clusters, and are processed into small RNAs 24–32 nucleotides (nt) in length. There are primarily 25- to 28-nt piRNAs in the testes of embryos [containing prospermatogonia (PSG)] and at <10 days postnatal [containing spermatogonia (SG) and spermatocytes before pachytene], whereas 28- to 30-nt piRNAs become abundant in testes at ≥10 days postnatal (containing SG, spermatocytes, and spermatids) (Aravin et al., 2007). Fetal piRNAs are enriched with repeat sequences, including retrotransposons, however, those in testes at >10 days postnatal are mostly derived from unique genomic sequences (Aravin et al., 2007; Kuramochi-Miyagawa et al., 2008).

**FIGURE 1:**
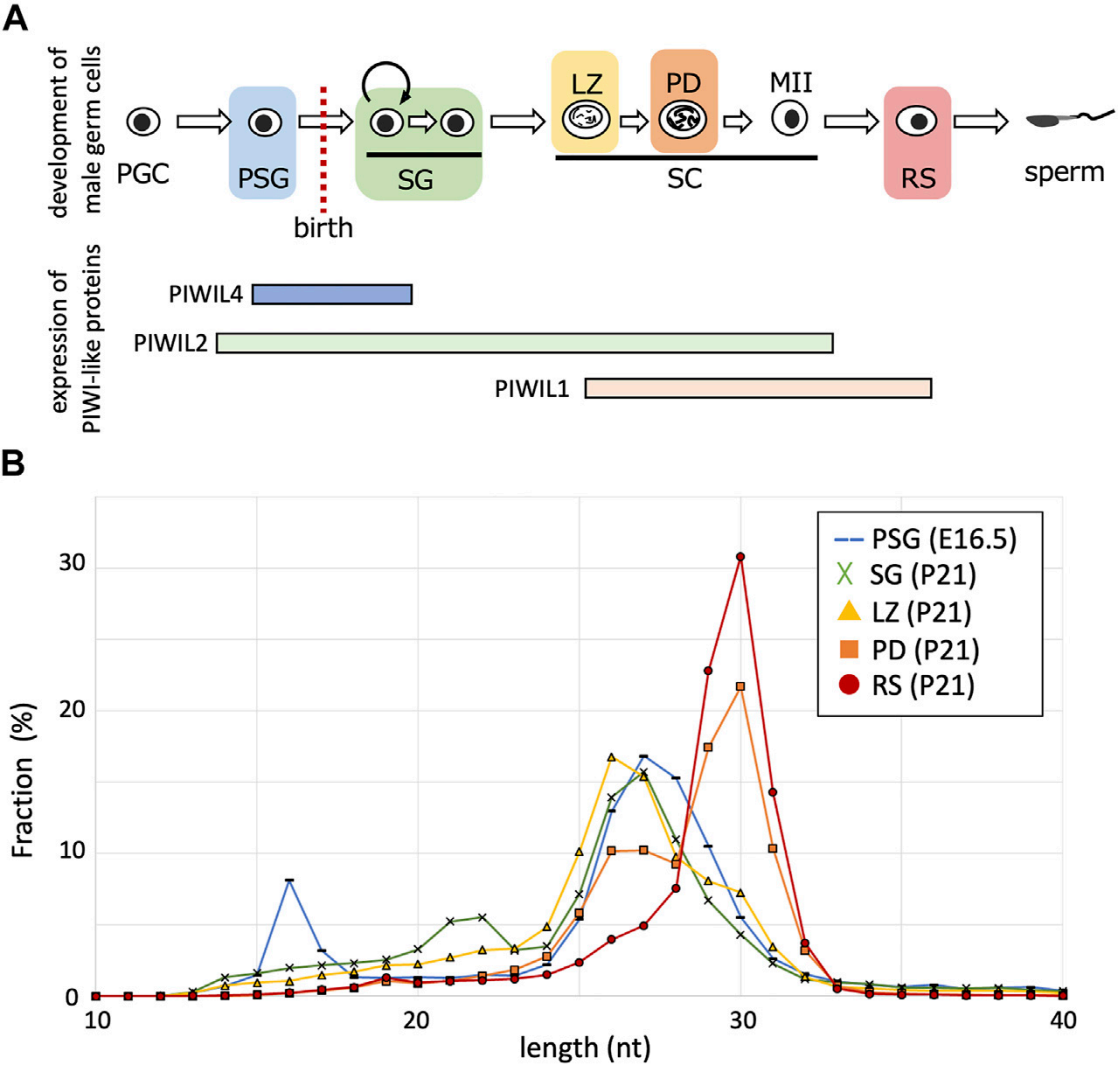
Length distributions of piRNA reads in developing germ cells. **(A)** The developmental order of male germ cells and the range of PIWI-like protein expression. Their expressions in postnatal germ cells are according to single-cell RNA-seq data (Hermann et al., 2018). PGC, primordial germ cell; PSG, prospermatogonium; SG, spermatogonium; SC, spermatocyte; LZ, leptotene and zygotene stage; PD, pachytene and diplotene stage; MII, meiosis II; RS, round spermatid. **(B)** Fractions (%) of piRNAs of indicated lengths (10–40 nt) in germ cells of different stages (PSG, blue; SG, green; LZ, yellow; PD, orange; RS, red).

PIWI-like proteins (PIWIL1, PIWIL2, and PIWIL4) belong to the Argonaute family with RNA cleavage activity. PIWIL2 is expressed in a wide range during germ cell development (PSG, SG, and spermatocytes), however, PIWIL4 expression is predominantly confined to PSG and undifferentiated SG, and PIWIL1 expression begins in meiotic spermatocytes (Figure 1A). Biochemical studies have revealed that PIWIL1, PIWIL2, and PIWIL4 bind to RNAs of 29–31 nt, 25–28 nt, and 26–29 nt in length, respectively (Kuramochi-Miyagawa et al., 2004; Aravin et al., 2008; Reuter et al., 2011). The PIWIL1 and PIWIL2 proteins cleave RNAs that are complementary to the bound piRNAs therefore, piRNAs complementary to retrotransposon mRNAs can guide their cleavage. The PIWIL2-cleaved RNAs are processed into secondary piRNAs in a process called ping-pong cycle, whereas the endonuclease activity of PIWIL4 is dispensable for the generation of piRNAs in PSG (De Fazio et al., 2011). Rather, the PIWIL4 binding to retrotransposon-derived piRNAs is involved in targeted *de novo* DNA methylation of retrotransposons in the genome (Carmell et al., 2007; Aravin et al., 2008; Kuramochi-Miyagawa et al., 2008).

The sequence features and roles of postnatal piRNAs, as well as dominant piRNA clusters, were studied using whole testis samples. However, testes contain germ cells at various developmental stages, including SG, spermatocytes, and spermatids. Therefore, it is important to analyze purified cell samples at each stage to understand the dynamics of piRNAs along the duration of germ cell development. Because of the development of a purification method for testicular germ cells by fluorescence-activated cell sorting (FACS) (Gaysinskaya et al., 2014), we previously sequenced piRNAs in various developmental stages, including SG, leptotene and zygotene spermatocytes (LZ), pachytene and diplotene spermatocytes (PD) and round spermatids (RS) (Inoue et al., 2017), as well as embryonic testes containing PSG (Ichiyanagi et al., 2014). Here, by in-depth analysis of these piRNAs in developing germ cells, we report the expression dynamics and features of piRNAs derived from piRNA clusters and retrotransposons.

## Results and Discussion

### Longer piRNAs (≧29 nt) Begin to be Expressed at Leptotene and Zygotene Spermatocytes

To characterize piRNAs expressed at various stages, we analyzed the small RNA sequencing data for PSG, SG, LZ, PD, and RS (Ichiyanagi et al., 2014; Inoue et al., 2017). The PSG data were obtained from samples at embryonic day 16.5, and the SG, LZ, PD, and RS data were for FACS-purified germ cells of the same individual at 21 days postnatal. After removing reads of miRNAs and cellular RNAs, such as rRNAs and tRNAs, the retained reads were mapped onto the mouse reference genome. The length distribution of the mapped piRNAs differed between the stages (Figure 1B). In PSG, the length peak was 27 nt, possibly reflecting a mixture of PIWIL2-and PIWIL4-bound piRNAs. In SG, the peak was shifted 1 nt shorter (26 nt), consistent with the predominance of PIWIL2-bound piRNAs. In LZ, the peak remained 26 nt, however, piRNAs of ≧29 nt became more abundant. In PD, the 26-nt peak became smaller, whereas the 30-nt peak became abundant. Eventually, in RS, only a single peak of 30 nt was observed, consistent with the predominance of PIWIL1-bound piRNAs. Therefore, shorter piRNAs were initially generated in male germ cells, and the population gradually shifted toward longer piRNAs during development. Notably, although long piRNAs are features of so-called “pachytene” piRNAs, our analysis revealed that such long piRNAs start to be generated at the LZ stages. To avoid ambiguity, we designated these longer piRNAs observed in spermatocytes and spermatids as “late piRNAs” (because they became expressed late) and shorter piRNAs in SG and spermatocytes as “early piRNAs” (because they were expressed early). As stated below, prospermatogonial piRNAs are distinct from these postnatal piRNAs; we designate these piRNAs as “PSG piRNAs”.

### The Expression Dynamics During Germ Cell Development Categorizes the piRNA Clusters Into Three Groups

**FIGURE 2:**
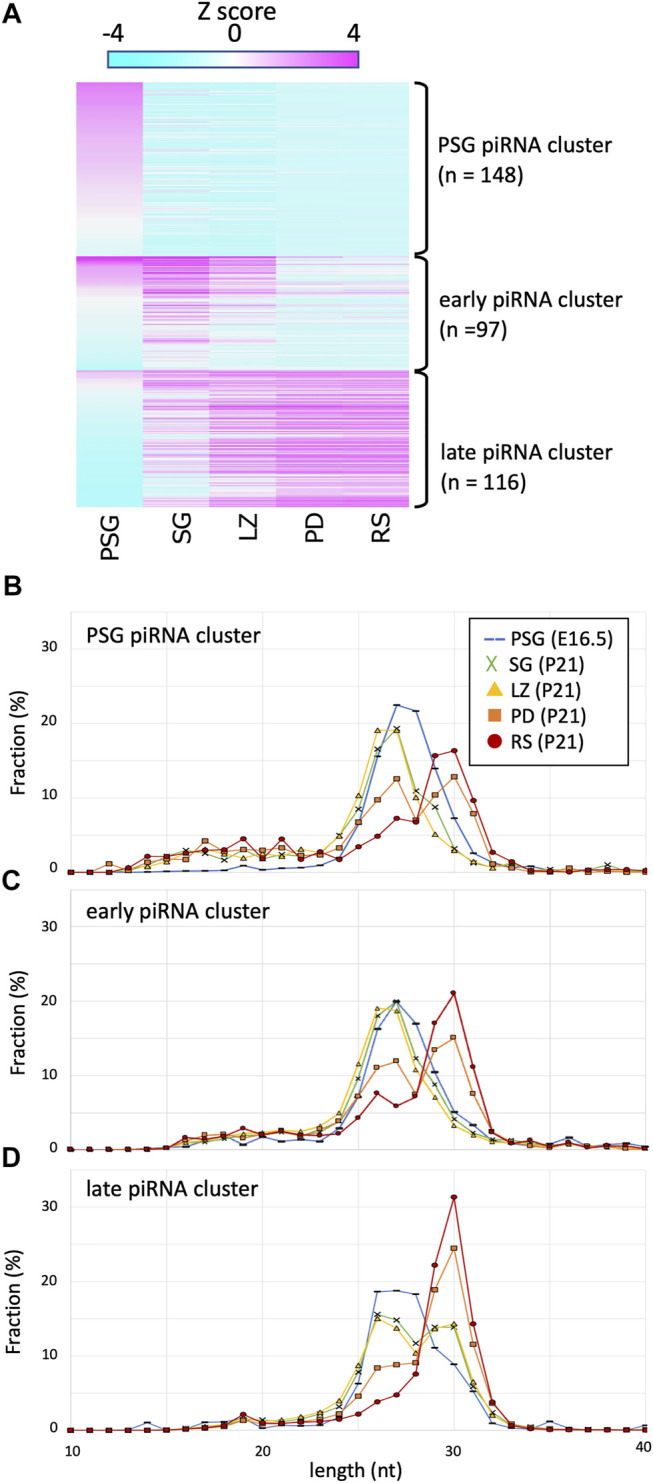
Expression profile and lengths of piRNA clusters. **(A)** A heatmap showing the piRNA expression levels for 512 piRNA clusters in different developmental stages. The clusters are ordered by the three piRNA categories: PSG, early, late piRNA clusters. **(B–D)** Length distributions of piRNAs derived from PSG **(B)**, early **(C)**, and late **(C)** piRNA clusters in the developmental stages. The color codes are the same as those in Figure 1B.

Certain piRNAs are generated *via* the ping-pong-cycle reactions for repeat-derived RNAs, whereas the majority are generated from precursor RNAs transcribed from piRNA clusters. To study the expression dynamics of cluster-derived piRNAs, we compiled piRNA clusters based on previous reports (Kuramochi-Miyagawa et al., 2008; Li et al., 2013) and piRNAdb (https://www.pirnadb.org), yielding a list of 512 piRNA clusters. The mapped reads in each developmental stage were counted and normalized by the genomic length of a cluster, yielding reads per kilobase per million mapped reads (RPKM values). K-means clustering revealed three major groups (Figure 2A; Supplementary Table S1): 1) clusters showing high expression in PSG but low expression in postnatal stages (PSG piRNA clusters), 2) clusters showing increased expression in SG and LZ but decreased expression in PD and RS (early piRNA clusters), and 3) clusters whose expression gradually increased as development proceeded (late piRNA clusters).

### The Lengths of piRNAs are Dictated by the PIWI-Like Proteins Expressed at Each Stage

Next, to gain mechanistic insights, we analyzed the lengths of piRNAs derived from each cluster at each developmental stage (Figures 2B–D). Regardless of the clusters from which they were derived, piRNA length was primarily determined by the developmental stage. For example, in PSG and SG, the piRNA lengths had a peak at approximately 27 nt, whereas the peak was at 30 nt in RS. It has been shown that piRNAs are processed by an endoribonuclease after loading onto PIWI proteins, and the structure of PIWI proteins determines the length of mature piRNAs that they bind (Kawaoka et al., 2011; Vourekas et al., 2012; Yamaguchi et al., 2020). The distribution of piRNA length is consistent with the idea that the relative number of PIWI-like proteins (PIWIL1 vs. PIWIL2) existing in the cell dictates the piRNA length. This implies that piRNAs of any cluster can bind to PIWIL1, PIWIL2, and PIWIL4 if these proteins are present in the cell.

However, we noticed that the PSG and early clusters generated shorter piRNAs than that of the late cluster in postnatal germ cells. In SG and LZ, the PSG and early clusters showed a single peak at approximately 27 nt (Figures 2B,C, green and yellow), whereas the late clusters showed a bimodal distribution with two peaks at 27 and 30 nt (Figure 2D, green and yellow). In PD, the PSG and early clusters showed two peaks at 27 and 30 nt (Figures 2B,C, orange), whereas the late clusters showed a predominant peak at 30 nt (Figure 2D, orange). In RS, the length of most piRNAs was approximately 30 nt (Figures 2B–D, red), however, a substantial fraction of piRNAs from the PSG and early clusters was approximately 27 nt in length (Figures 2B,C, red). These results suggest that piRNAs of each cluster have a preference for PIWI-like proteins to bind. The sequence features of precursor RNAs may be involved in the difference in preference between the PSG/early and late clusters.

### Late piRNA Clusters Were Expressed in LZ Spermatocytes in the Second and Later Wave, but not in the First Wave of Spermatogenesis

**FIGURE 3:**
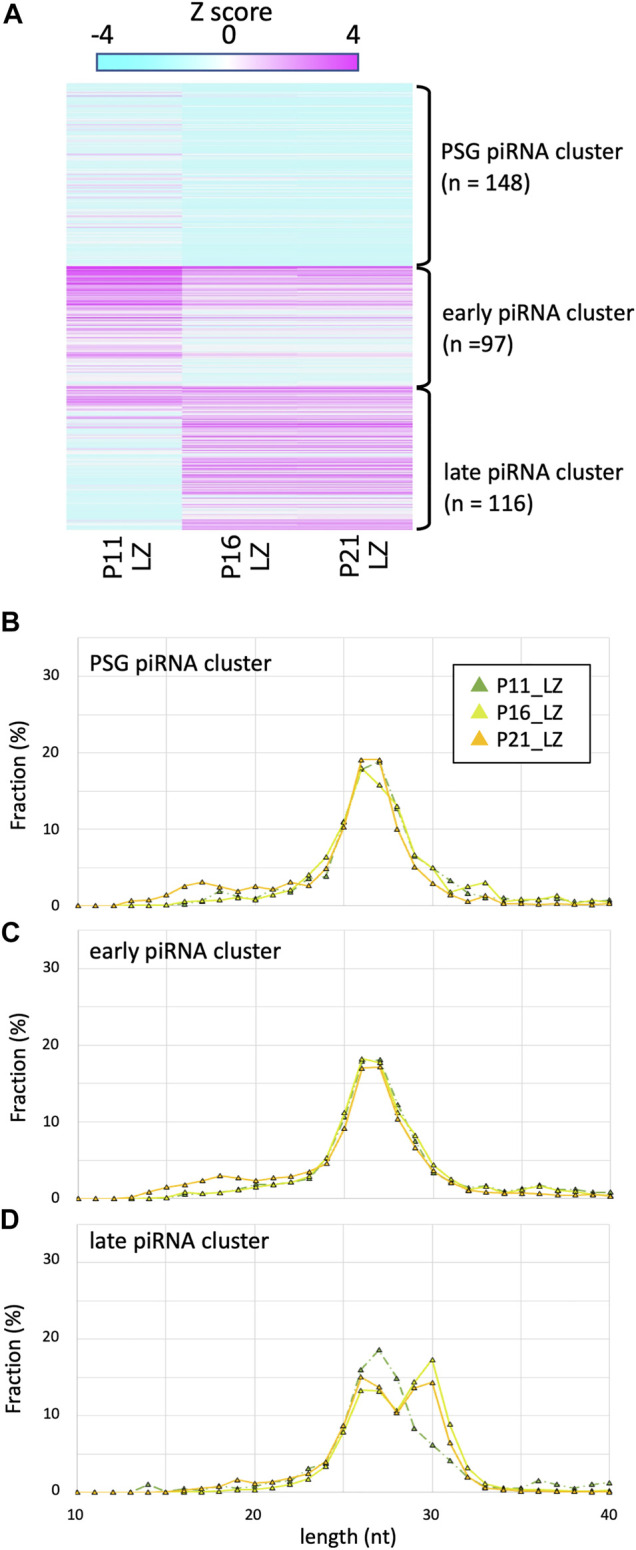
Expression profile and lengths of piRNA clusters in P11, P16, and P21 LZ spermatocytes **(A)** A heatmap showing the piRNA expression levels for 512 piRNA clusters in different developmental stages. **(B–D)** Length distributions of piRNAs derived from PSG **(B)**, early **(C)**, and late **(C)** piRNA clusters. The color codes are the same as those in Figure 1B.

It has been shown that piRNAs in testes at P10 are predominantly 25–28 nt in length (Aravin et al., 2007). Since such testes contain spermatogonia and preleptotene, leptotene and zygotene spermatocytes (but not germ cells of pachytene and later stages), these piRNAs are called pre-pachytene piRNAs. This seemed inconsistent with our data for P21 LZ spermatocytes, expressing longer piRNAs, which were derived from late piRNA clusters. It is conceivable that LZ spermatocytes at different ages produce piRNAs of different lengths. Therefore, we prepared LZ spermatocytes from P11 and P16 testes by cell sorting (Supplementary Figure S1) as for P21 testes (Inoue et al., 2017), and piRNAs in the LZ samples were compared (Figure 3). In P16 LZ spermatocytes, the expression levels of late piRNA clusters were almost the same, and the piRNA length distribution was also very similar. In P11 LZ spermatocytes, however, expression levels of late piRNA clusters were much lower than those in P16 and P21 LZ spermatocytes.

P11 testes do not contain pachytene spermatocytes, whereas P16 and P21 do. However, the presence of longer piRNAs in P16 and P21 LZ preparations cannot be explained by contamination, if any, of PD spermatocytes because of the high purity (>85%) of the cell fractions (Gaysinskaya et al., 2014; Inoue et al., 2017). LZ spermatocytes at P11 are developmentally distinct, as they are produced in the first wave of spermatogenesis, where spermatocytes are differentiated from non-stem-cell spermatogonia (Yoshida et al., 2006). On the other hand, at P16 and later, spermatocytes are differentiated from spermatogonial stem cells. These suggest that the first-wave LZ spermatocytes very weakly express the late piRNA cluster, whereas LZ spermatocytes in subsequent waves start to express these piRNAs. The LZ spermatocytes express *Piwil1* (Hermann et al., 2018), which is consistent with the presence of PIWIL1-type longer piRNAs.

### Differential Expression of Retrotransposon-Derived piRNAs During Germ Cell Development

**FIGURE 4:**
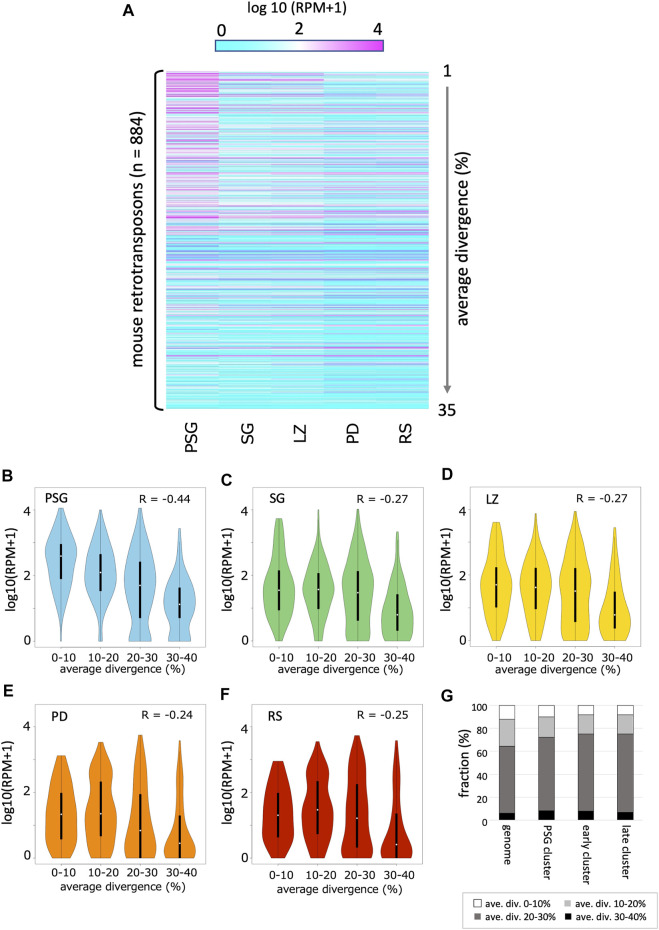
Expression profile of retrotransposon-derived piRNAs **(A)** A heatmap showing the piRNA expression levels for 884 retrotransposon species identified in the mouse genome (by RepeatMasker). The order of retrotransposons is sorted by the mean values of divergence from the respective consensus sequences. The evolutionally younger a family is, the smaller its value is. **(B–F)** Violin plots showing the expression levels of retrotransposons categorized by their average divergences in PSG **(B)**, SG **(C)**, LZ **(D)**, PD **(E)** and RS **(F)**. In each panel, the first (0–10%), second (10–20%), third (20–30%), and fourth (30–40%) groups contain 90, 249, 377, and 168 retrotransposons, respectively. **(G)** Retrotransposon contents in PSG, early, and late piRNA clusters as well as whole genome. Retrotransposons are grouped according to their average divergences.

As expected, a substantial portion of piRNAs were mapped onto the retrotransposon sequences in the genome. Thus, we analyzed the time course of the abundance of retrotransposon-derived piRNAs (Figure 4A; Supplementary Table S2). In general, the expression of retrotransposon-derived piRNAs was highest in PSG, and gradually declined as development proceeded, which is consistent with the fact that piRNAs in PSG mainly work for post-transcriptional regulation and *de novo* DNA methylation of retrotransposons (Inoue et al., 2017).

Next, we analyzed the relationship between the evolutionary ages of retrotransposons and piRNA abundance. The average divergence from the respective consensus sequences was used to estimate the age of retrotransposons in the genome. In PSG, younger retrotransposons (with lower divergence) had more piRNAs (Figure 4B, *R* = –0.44, *p* < 10^–15^). PSG piRNA clusters did not show an enrichment of young retrotransposon copies (Figure 4G). Thus, the high abundance of piRNAs from young retrotransposons is likely due to the relatively high expression of their mRNAs in prospermatogonia, which can fuel the ping-pong cycle amplification of piRNAs. This is also consistent with the role of piRNAs in the host defense system against the mobility of active retrotransposons in PSG. However, in SG and the later developmental stages, the relationship between age and piRNA abundance became weaker (Figures 4C–F).

### Conclusion: New Terminologies of Male Germline piRNA Clusters Based on Their Gradual Changes During Meiosis

Here, we compared the features of piRNAs expressed at various stages of male germ cell development in mice. “Pachytene piRNAs” observed in testes at ages 10 days and older are mainly long (29–31 nt) piRNAs, whereas “pre-pachytene piRNAs” observed in testes at ages 9 days and younger are mainly short (25–28 nt) piRNAs (Aravin et al., 2007). However, the present study revealed that, although longer piRNAs are highly expressed in RS, substantial amounts are also expressed at earlier stages, such as SG and LZ, particularly for piRNAs derived from the late piRNA clusters (Figure 2D). Likewise, characteristic “pre-pachytene piRNAs” (those from the PSG and early clusters) remain at the pachytene and later (PD and RS; Figures 2B,C). Since the length distribution and expression abundance gradually change in developmental stages, from SG *via* LZ and PD to RS (Figures 1, 2), it is inappropriate to categorize piRNAs (and clusters) into pachytene and pre-pachytene piRNAs based on their expression levels in whole testes. Here, we propose to divide them into three categories based on their expression patterns in male germ cells: PSG, early, and late piRNA clusters (see Supplementary Table S1 for details).

## Materials and Methods

### Small RNA Sequencing Library Preparation

Leptotene and zygotene spermatocytes were prepared from mouse testes at postnatal days 11 and 16 (3 individuals respectively) according to the published protocol (Gaysinskaya et al., 2014; Inoue et al., 2017), with an exception that SH800 (SONY) was used for cell sorting (Supplementary Figure S1). RNAs were prepared from the sorted cells by isogene (Nippongene) and DirectoZol RNA prep kit (Zymo Research), quantified by Qubit (Thermo Fisher), and analyzed by TapeStation (Agilent technology). Small RNA-seq libraries were prepared from 31 to 23 ng of total RNAs (of P11 and P16, respectively) using the NEBNext Small RNA library Prep set (NEB). The libraries were sequenced on Illumina MiSeq in a 60-bp single-end run (yielding 14 million reads per sample, respectively). The sequencing data has been deposited to Gene Expression Omnibus (GEO) under the accession number, GSE200853.

### Data Analysis

The published dataset of small RNA sequencing (50-bp single-end sequencing) was retrieved from the Gene Expression Omnibus under accession numbers GSE70891 (for PSG at embryonic day 16.5) and GSE54515 (for SG, LZ, PD, and RS at 21 days postnatal). After clipping the adapter sequence, the reads were mapped to miRNAs, rRNAs, tRNAs, snoRNAs, snRNAs, and scRNAs by Bowtie2 (Langmead and Salzberg, 2012) without allowing mismatches using the following options: L 10 --norc--score-min L,0,0 --no-unal. The mapped reads were discarded, and unmapped reads were then mapped to the mouse reference genome (mm10) using Bowtie2 without allowing mismatches using the options “-L 10 --score-min L,0,0”. For the analysis of cluster-derived piRNAs, we extracted uniquely mapped reads and analyzed their lengths (10–40 bp).

### The piRNA Cluster List

The piRNA clusters used in the present study were obtained from published papers (Kuramochi-Miyagawa et al., 2008; Li et al., 2013) and piRNAdb (https://www.pirnadb.org/), and genomic coordinates were converted to mm10 using LiftOver obtained from the UCSC genome browser (Navarro Gonzalez et al., 2021). All clusters were merged using Bedtools with option -d 100, yielding 512 clusters.

### Calculation of piRNA Expression From Clusters and Retrotransposons

To calculate the piRNA expression from clusters, uniquely mapped reads were counted for each cluster and normalized by the length of the cluster and the total number of mapped reads in the library (RPKM). The clusters were grouped based on the log expression of piRNAs at each stage using the k-means clustering method.

To calculate the expression of the retrotransposon-derived piRNAs, reads were mapped onto the mouse genome (mm10), and the RepeatMasker annotation (Navarro Gonzalez et al., 2021) of these regions was used to identify repeat-derived reads. Both sense and antisense piRNAs were counted. To estimate the ages of retrotransposons, the miliDiv values in the RepeatMasker track (per mille divergence from the consensus sequence) were averaged for all genomic copies belonging to respective retrotransposon subfamilies (or sub-subfamilies) that were annotated in the repName column.

## Data Availability

The datasets presented in this study can be found in online repositories. The names of the repository/repositories and accession number(s) can be found in the article/Supplementary Material.

## References

[B1] Aravin A. A. Sachidanandam R. Bourc'his D. Schaefer C. Pezic D. Toth K. F. (2008). A piRNA Pathway Primed by Individual Transposons Is Linked to De Novo DNA Methylation in Mice. Mol. Cell. 31 (6), 785–799. 10.1016/j.molcel.2008.09.003 18922463 PMC2730041

[B2] Aravin A. A. Sachidanandam R. Girard A. Fejes-Toth K. Hannon G. J. (2007). Developmentally Regulated piRNA Clusters Implicate MILI in Transposon Control. Science 316 (5825), 744–747. 10.1126/science.1142612 17446352

[B3] Carmell M. A. Girard A. van de Kant H. J. G. Bourc'his D. Bestor T. H. de Rooij D. G. (2007). MIWI2 Is Essential for Spermatogenesis and Repression of Transposons in the Mouse Male Germline. Dev. Cell. 12 (4), 503–514. 10.1016/j.devcel.2007.03.001 17395546

[B4] Chuma S. Nakano T. (2013). piRNA and Spermatogenesis in Mice. Phil. Trans. R. Soc. B 368 (1609), 20110338. 10.1098/rstb.2011.0338 23166399 PMC3539364

[B5] De Fazio S. Bartonicek N. Di Giacomo M. Abreu-Goodger C. Sankar A. Funaya C. (2011). The Endonuclease Activity of Mili Fuels piRNA Amplification that Silences LINE1 Elements. Nature 480 (7376), 259–263. 10.1038/nature10547 22020280

[B6] Gaysinskaya V. Soh I. Y. van der Heijden G. W. Bortvin A. (2014). Optimized Flow Cytometry Isolation of Murine Spermatocytes. Cytometry 85 (6), 556–565. 10.1002/cyto.a.22463 24664803 PMC4246648

[B7] Hermann B. P. Cheng K. Singh A. Roa-De La Cruz L. Mutoji K. N. Chen I.-C. (2018). The Mammalian Spermatogenesis Single-Cell Transcriptome, from Spermatogonial Stem Cells to Spermatids. Cell. Rep. 25 (6), 1650–1667. 10.1016/j.celrep.2018.10.026 30404016 PMC6384825

[B8] Ichiyanagi T. Ichiyanagi K. Ogawa A. Kuramochi-Miyagawa S. Nakano T. Chuma S. (2014). HSP90α Plays an Important Role in piRNA Biogenesis and Retrotransposon Repression in Mouse. Nucleic Acids Res. 42 (19), 11903–11911. 10.1093/nar/gku881 25262350 PMC4231750

[B9] Inoue K. Ichiyanagi K. Fukuda K. Glinka M. Sasaki H. (2017). Switching of Dominant Retrotransposon Silencing Strategies from Posttranscriptional to Transcriptional Mechanisms during Male Germ-Cell Development in Mice. PLoS Genet. 13 (7), e1006926. 10.1371/journal.pgen.1006926 28749988 PMC5549759

[B10] Kawaoka S. Izumi N. Katsuma S. Tomari Y. (2011). 3′ End Formation of PIWI-Interacting RNAs *In Vitro* . Mol. Cell. 43 (6), 1015–1022. 10.1016/j.molcel.2011.07.029 21925389

[B11] Kuramochi-Miyagawa S. Kimura T. Ijiri T. W. Isobe T. Asada N. Fujita Y. (2004). Mili, a Mammalian Member Ofpiwifamily Gene, Is Essential for Spermatogenesis. Development 131 (4), 839–849. 10.1242/dev.00973 14736746

[B12] Kuramochi-Miyagawa S. Watanabe T. Gotoh K. Totoki Y. Toyoda A. Ikawa M. (2008). DNA Methylation of Retrotransposon Genes Is Regulated by Piwi Family Members MILI and MIWI2 in Murine Fetal Testes. Genes. Dev. 22 (7), 908–917. 10.1101/gad.1640708 18381894 PMC2279202

[B13] Langmead B. Salzberg S. L. (2012). Fast Gapped-Read Alignment with Bowtie 2. Nat. Methods 9 (4), 357–359. 10.1038/nmeth.1923 22388286 PMC3322381

[B14] Li X. Z. Roy C. K. Dong X. Bolcun-Filas E. Wang J. Han B. W. (2013). An Ancient Transcription Factor Initiates the Burst of piRNA Production during Early Meiosis in Mouse Testes. Mol. Cell. 50 (1), 67–81. 10.1016/j.molcel.2013.02.016 23523368 PMC3671569

[B15] Navarro Gonzalez J. Zweig A. S. Speir M. L. Schmelter D. Rosenbloom K. R. Raney B. J. (2021). The UCSC Genome Browser Database: 2021 Update. Nucleic Acids Res. 49 (D1), D1046–D1057. 10.1093/nar/gkaa1070 33221922 PMC7779060

[B16] Reuter M. Berninger P. Chuma S. Shah H. Hosokawa M. Funaya C. (2011). Miwi Catalysis Is Required for piRNA Amplification-independent LINE1 Transposon Silencing. Nature 480 (7376), 264–267. 10.1038/nature10672 22121019

[B17] Vourekas A. Zheng Q. Alexiou P. Maragkakis M. Kirino Y. Gregory B. D. (2012). Mili and Miwi Target RNA Repertoire Reveals piRNA Biogenesis and Function of Miwi in Spermiogenesis. Nat. Struct. Mol. Biol. 19 (8), 773–781. 10.1038/nsmb.2347 22842725 PMC3414646

[B18] Yamaguchi S. Oe A. Nishida K. M. Yamashita K. Kajiya A. Hirano S. (2020). Crystal Structure of Drosophila Piwi. Nat. Commun. 11 (1), 858. 10.1038/s41467-020-14687-1 32051406 PMC7015924

[B19] Yoshida S. Sukeno M. Nakagawa T. Ohbo K. Nagamatsu G. Suda T. (2006). The First Round of Mouse Spermatogenesis Is a Distinctive Program that Lacks the Self-Renewing Spermatogonia Stage. Development 133 (8), 1495–1505. 10.1242/dev.02316 16540512

