# Supplementary figures and images for: The Expression Dynamics of piRNAs Derived From Male Germline piRNA Clusters and Retrotransposons
Source: Front Cell Dev Biol. 2022 May 11;10:868746. doi: 10.3389/fcell.2022.868746 (PMC9130748; doi:10.3389/fcell.2022.868746)

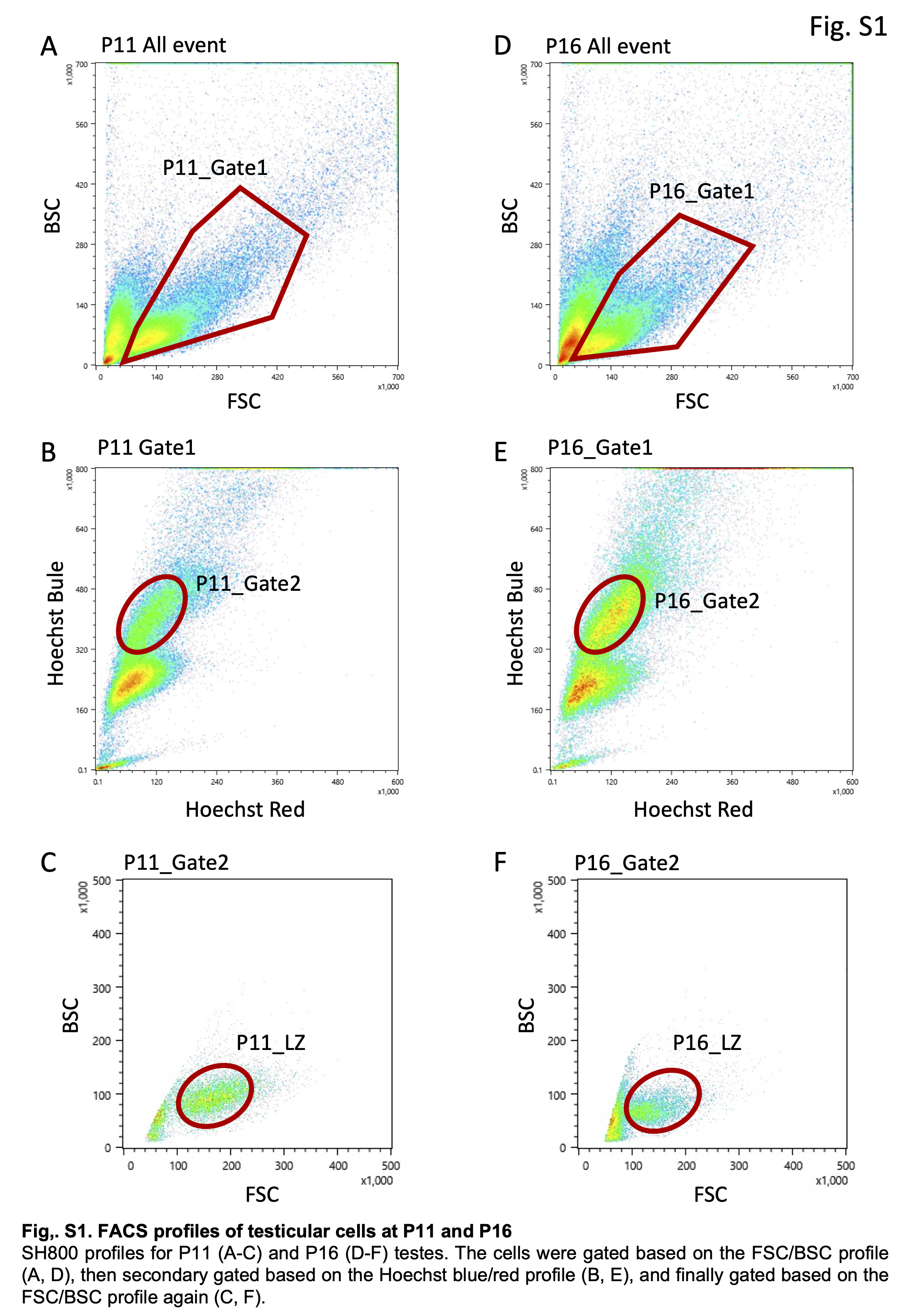

Supplement: Supplementary file 1 [file Image1.TIFF]
